# Inferring a nonlinear biochemical network model from a heterogeneous single-cell time course data

**DOI:** 10.1038/s41598-018-25064-w

**Published:** 2018-05-01

**Authors:** Yuki Shindo, Yohei Kondo, Yasushi Sako

**Affiliations:** 1 Cellular Informatics Laboratory, RIKEN, Wako, Saitama 351-0198 Japan; 2 Graduate School of Informatics, Kyoto University, Sakyo-ku, Kyoto 606-8501 Japan

## Abstract

Mathematical modeling and analysis of biochemical reaction networks are key routines in computational systems biology and biophysics; however, it remains difficult to choose the most valid model. Here, we propose a computational framework for data-driven and systematic inference of a nonlinear biochemical network model. The framework is based on the expectation-maximization algorithm combined with particle smoother and sparse regularization techniques. In this method, a “redundant” model consisting of an excessive number of nodes and regulatory paths is iteratively updated by eliminating unnecessary paths, resulting in an inference of the most likely model. Using artificial single-cell time-course data showing heterogeneous oscillatory behaviors, we demonstrated that this algorithm successfully inferred the true network without any prior knowledge of network topology or parameter values. Furthermore, we showed that both the regulatory paths among nodes and the optimal number of nodes in the network could be systematically determined. The method presented in this study provides a general framework for inferring a nonlinear biochemical network model from heterogeneous single-cell time-course data.

## Introduction

A biochemical reaction network is a key concept in understanding how higher-order functions in the cell emerge from relatively simple individual elements, such as proteins and metabolites. The reaction network system is often nonlinear and complex and can potentially display various dynamic behaviors, such as ultrasensitivity, bistability, and oscillation^1–6^, that form the basis of diverse cellular phenotypes. Because of its complexity, *in silico* analysis based on mathematical modeling and numerical simulation is an essential strategy for quantitatively understanding a system of interest. Mathematical analysis can help to eliminate the nonessential individuality of biological targets and identify core principles that govern the behaviors and function of the system in the cell. Using these approaches, various studies have revealed relationships between the behavior of a system and its underlying mechanisms, including feedback/feedforward loops, cross-talk, compartmentalization, and noise^7–12^.

There are at least two distinct stages of *in silico* network analysis. The first step involves construction of a mathematical model that describes the system, and the second step involves analysis of the model. Although the second step strongly depends upon the aim of the study, a mathematical model is needed for the analysis, regardless of the details of the second step. Typically, modeling of a target system is performed in a patchwork manner, which means that fragments of studies regarding a specific reaction are integrated to construct a map of the reaction network^13–16^. Although this procedure is straightforward, selecting the sources of each reaction that constitutes the network is a non-trivial task that might raise concerns regarding the validity of the modeling. Alternatively, a data-driven approach incorporating as few assumptions as possible for inferring a network model can compensate for the defect of the patchwork modeling.

Data-driven inference of biochemical network models has previously been extensively studied^17–20^, and both genome-wide networks and cell-specific gene regulatory and posttranslational modification networks have been systematically reconstructed^21,22^. Additionally, although the regulatory relationships in the inferred network often represent linear or binary correlations among nodes, efforts are underway to identify nonlinear ordinary differential equation (ODE) systems^23–26^. However, the intersection between systematic model inference and network modeling with nonlinear ODEs has received less attention^27,28^. Therefore, a framework that enables data-driven modeling of the network while considering the nonlinearity of the system is needed. Furthermore, recent advances in experimental methods have made available highly quantitative and time-resolved data at the single-cell level^29^, thereby making it desirable for the framework to handle single-cell datasets.

**Figure 1:**
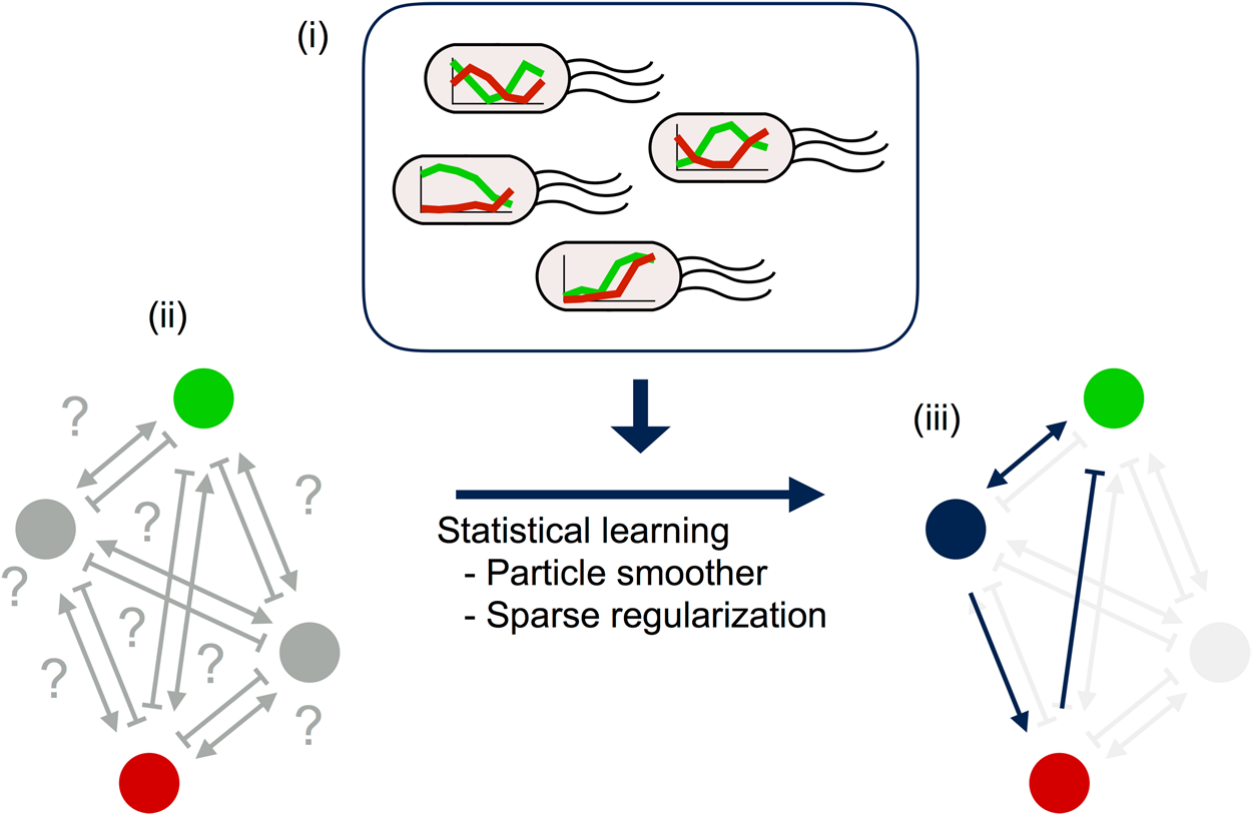
Schematic representation of the proposed method for data-driven inference of biochemical network models. Details of each step are described in the text.

To address these problems, we developed a method combining an expectation-maximization (EM) algorithm with a particle smoother and sparse regularization. Using this method, we showed that an oscillatory network model can be systematically inferred based only on single-cell time-course data. Briefly, our strategy is as follows (Fig. 1): (1) quantitatively measure components of the network and obtain a single-cell dataset, (2) prepare a “redundant” model where an excessive number of reaction paths and nodes are defined using nonlinear ODEs, and (3) perform model learning using the dataset while eliminating unnecessary paths in the redundant model to identify the most probable model. We evaluated the performance of the method using artificial time-course data and showed that the algorithm accurately inferred the true network model in a data-driven manner.

## Results

### Maximum likelihood parameter estimation in a biochemical network model

We introduced the following nonlinear state space model:

1 $$\begin{array}{rcl}{{\boldsymbol{x}}}_{t} & = & {\boldsymbol{f}}({{\boldsymbol{x}}}_{t-1})+{{\boldsymbol{v}}}_{t}\\ {{\boldsymbol{y}}}_{t} & = & {\boldsymbol{h}}({{\boldsymbol{x}}}_{t})+{{\boldsymbol{w}}}_{t}\end{array}$$

where ***x*** and ***y*** denote state variables (e.g., amounts of mRNA and protein) and measurements (e.g., fluorescence intensity), respectively. Function ***f*** describes the evolution of the system and can be calculated as ${\boldsymbol{f}}({{\boldsymbol{x}}}_{t-1})={{\boldsymbol{x}}}_{t-1}+{\int }_{t-1}^{t}{\boldsymbol{g}}({{\boldsymbol{x}}}_{\tau },{{\boldsymbol{\theta }}}_{sys})d\tau$, where, in general, ***g*** represents ODEs that describe a biochemical reaction network of interest, and ***θ***_*sys*_ denotes model parameters. Function ***h*** represents the process of measurement of ***x***. Vectors ***v***_*t*_ and ***w***_*t*_ denote system noise and measurement noise, respectively, where we assumed that they followed a Gaussian distribution. Given dataset $Y=\{{Y}^{(a)}\}(a=1,\ldots ,A)$, where *a* is an index of each cell and *Y*^(*a*)^ represents the single-cell time-course data, estimation of ***θ***, a set of parameters that characterize the state space model, can be accomplished by maximizing the log-likelihood of the model. Here, we employed an EM algorithm to find the maximum likelihood estimates of ***θ***. Note that the algorithm is analytically intractable, because it requires a probability distribution of the time course at all time points. Therefore, we numerically approximated the probability distribution using a particle smoother algorithm^30^ (Materials and Methods). We referred to the algorithm^31,32^ as the EM-PS (particle smoother) algorithm.

**Figure 2:**
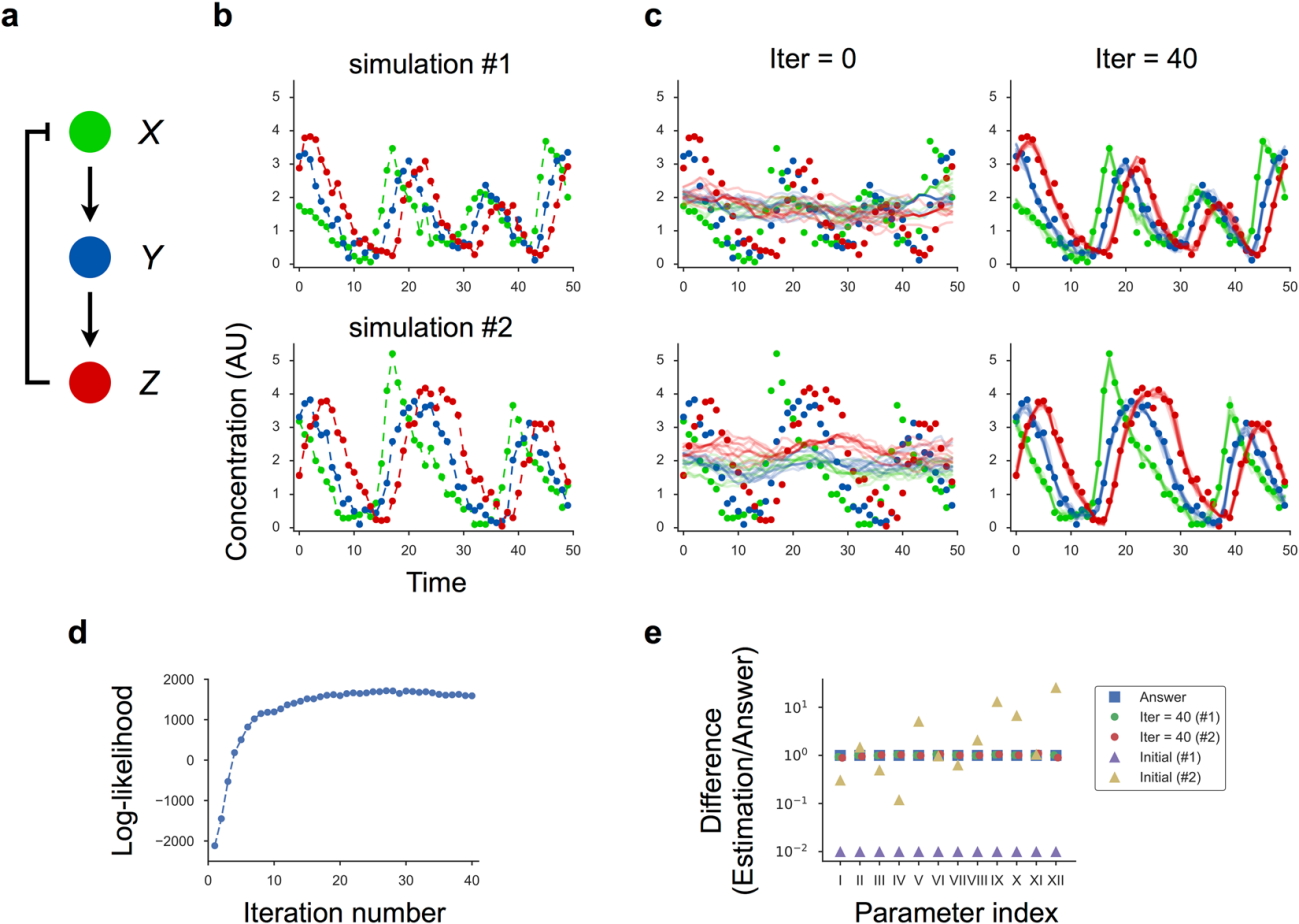
Maximum likelihood estimation of model parameters using the EM-PS algorithm. (**a**) Schematic of a three-component negative feedback oscillator model. (**b**) Artificial measurement data were generated by numerically solving the model as nonlinear stochastic Langevin equations, followed by addition of Gaussian noise to each value to simulate the measurement process. The dataset consists of 10 independent time-course data, with two examples (#1 and #2) shown. (**c**) Iterative estimation of the states using the EM-PS algorithm. Each dot represents the (artificial) measurement data, and lines denote the trajectories sampled by the particle smoother. (**d**) Log-likelihood values were plotted as a function of the iteration number. Note that small fluctuations were observed, even after the convergence of the algorithm because of the stochastic nature of the EM-PS algorithm. (**e**) A difference in the parameter values between the estimated and correct values is shown as a ratio. We tested two different sets of initial parameter values, where one is 1/100 of the correct values (Initial #1), and another is randomly generated in the range of 1/30 to 30× the correct values (Initial #2).

Next, we tested whether the EM-PS algorithm could provide correct estimates in a given model using artificial time-course data. To generate artificial data, we constructed a gene regulatory network *in silico* that consisted of three genes (*X*, *Y*, and *Z*) and a negative feedback loop (Fig. 2a). The network produced an oscillatory expression pattern with appropriate parameters. We used the Hill function to express reactions involving either activator or repressor molecules, because the activity of such regulators is often nonlinear (Supplementary Information). For simplicity, we used first-order kinetics for the degradation process. To mimic a realistic biological experiment in which cell-to-cell variability and observation noise exist, we numerically solved the model as nonlinear stochastic Langevin equations and added Gaussian noise as observation error to each value to generate artificial single-cell time-course data (Fig. 2b).

Using the artificial data and EM-PS algorithm, we conducted maximum likelihood estimation of the model parameters. We observed a monotonic increase in the log-likelihood during iterations of the algorithm, and eventually the estimated states were consistent with the data (Figs 2c and d and S1). Note that small fluctuations were observed, even after convergence due to the stochastic nature of the particle smoother algorithm implemented in the EM-PS algorithm. Differences between the correct and estimated values were <11%, even though the dataset contained significant cell-to-cell variability. Additionally, the algorithm was robust over a wide range of initial values (Fig. 2e; see Initial #1 and #2). Interestingly, the dynamics of parameter convergence varied among parameters and were not always monotonic (Supplementary Fig. S2), indicating that the likelihood function had a complicated landscape in the parameter space. These results revealed that the EM-PS algorithm represented a powerful approach for parameter estimation in nonlinear biochemical network models.

### Inferring network topology using a sparse regularized EM-PS algorithm

Next, we extended the algorithm to infer not only parameter values but also network topology. We focused on the fact that biochemical networks are sparse^33–35^, which means that the number of regulatory paths is much smaller than the number of possible links between nodes. To utilize the sparsity of biochemical networks for inference^35–37^, we introduced a regularization term referred to as the least absolute shrinkage and selection operator (Lasso), which is a simple yet powerful technique that provides a sparse solution^38^. In our strategy, we first prepared a “redundant” model consisting of an excessive number of regulatory paths among genes, followed by elimination of less important paths by Lasso.

To construct the redundant model, we used the Hill function, because it can express both a linear and nonlinear reaction depending on parameters *K* and *n*, which denote the (apparent) association constant [reciprocal of the (apparent) dissociation constant] and Hill coefficient, respectively. For example, the activity of transcription activator, *A*, or repressor, *R*, was expressed as ${c}_{A}={(K[A])}^{n}/(1+{(K[A])}^{n}),\,{c}_{R}=1/(1+{(K[R])}^{n})$. Assuming a common situation in which regulators function independently^39^, overall gene expression that is regulated by virtually any gene in the system (redundant model) can be written as follows:

2 $${\rm{production}}\,{\rm{rate}}=(\sum _{i}\,{a}_{i}\frac{{({K}_{i}[{X}_{i}])}^{{n}_{i}}}{1+{({K}_{i}[{X}_{i}])}^{{n}_{i}}})\cdot \prod _{j}\frac{1}{1+{({K}_{-j}[{X}_{j}])}^{{n}_{j}}}$$

where *i* and *j* represent indices of the activator and repressor, respectively. We focused on the fact that a path does not exist [*c*_*A*_ = 0 (no activator activity) or *c*_*R*_ = 1 (no repressor activity)] when parameter *K* = 0 (i.e., a zero or nonzero association constant can be used to characterize the presence or absence of the regulatory path in the model). Using this notation, the condition that biochemical networks are sparse is equivalent to the fact that most association constants (*K*) in the redundant model are equal to zero. Therefore, the association constants were subjected to regularization, thereby virtually removing the less important path from the network. Therefore, we rewrote the equations regarding the EM steps as follows:

3 $$\begin{array}{rcl}Q^{\prime} ({\boldsymbol{\theta }},{{\boldsymbol{\theta }}}^{({\rm{old}})}) & = & Q({\boldsymbol{\theta }},{{\boldsymbol{\theta }}}^{({\rm{old}})})-\,\lambda \sum _{s}|{K}_{s}|\\ {{\boldsymbol{\theta }}}^{({\rm{new}})} & = & {\rm{\arg }}\,\mathop{{\rm{\max }}}\limits_{{\boldsymbol{\theta }}}Q^{\prime} ({\boldsymbol{\theta }},{{\boldsymbol{\theta }}}^{({\rm{old}})})\end{array}$$

where *s* represents the index of the association constant, and *λ* denotes the strength of the regularization term (details are provided in Materials and Methods). We referred to this algorithm as the EM-PS-Lasso algorithm.

**Figure 3:**
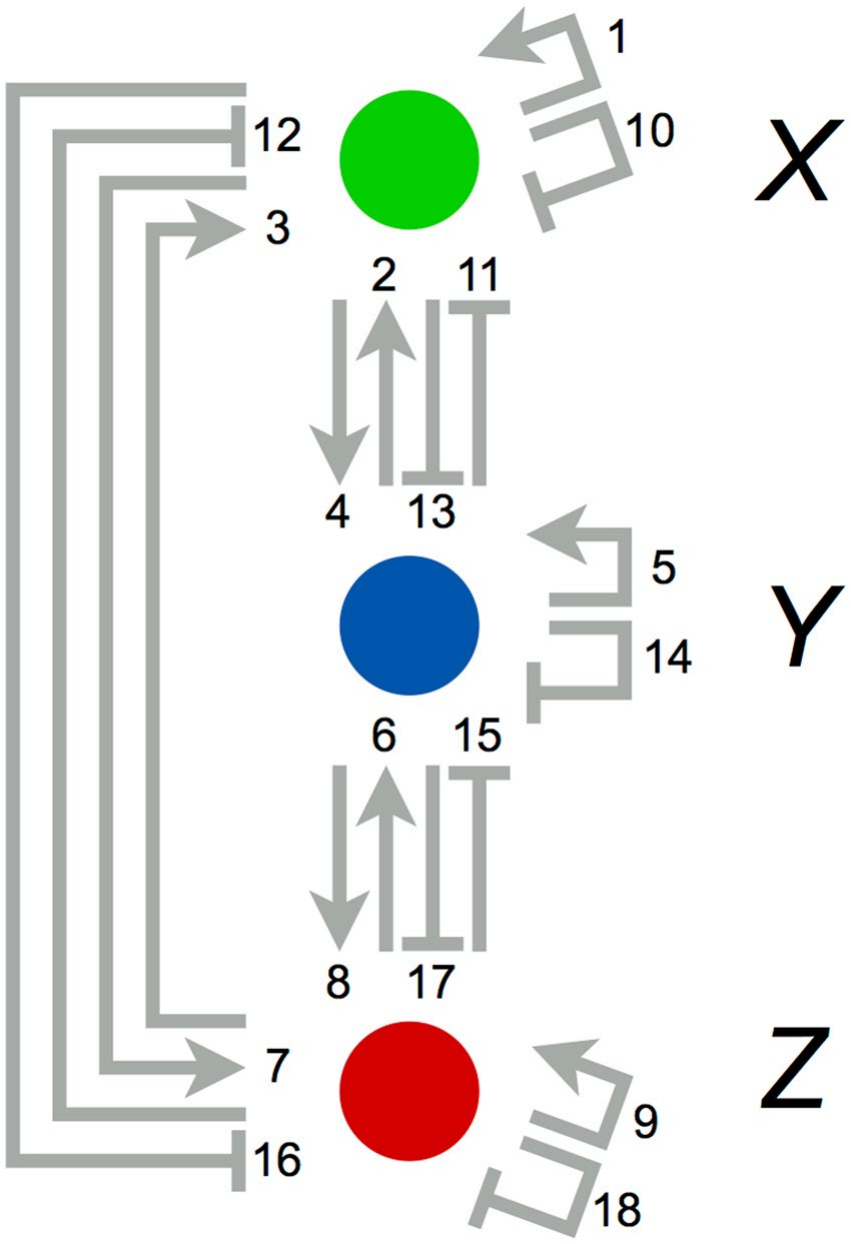
Schematic of the redundant model. We assumed no prior knowledge regarding the regulatory relationships in the network. Therefore, the model consists of an excessive number of regulatory paths among genes. The numbers shown in the network scheme represent the index of each reaction path.

**Figure 4:**
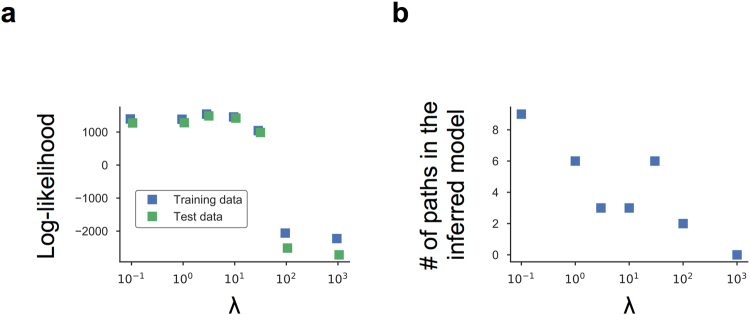
Inferring the network model via the EM-PS-Lasso algorithm. (**a**) The models were inferred using the EM-PS-Lasso algorithm, with different values for the regularization parameter, *λ*, and using the artificial data and redundant model. Log-likelihood values at iteration number 100 were plotted as a function of *λ*. (**b)** Relationship between the number of effective paths in the inferred models and *λ*.

We then tested the performance of the EM-PS-Lasso algorithm using artificial data (Fig. 2b). We assumed a situation where we had time-course data for genes *X*, *Y*, and *Z*, but no prior knowledge of their regulatory relationships. Therefore, we constructed a model where any possible regulatory paths among the three genes (18 paths) were incorporated (Fig. 3 and Supplementary Information). Using the redundant model and artificial single-cell time-course data, we conducted network inference and parameter estimation using the EM-PM-Lasso algorithm. Because this algorithm requires parameter *λ*, which controls the strength of the penalty term, we evaluated the log-likelihood of the model as a function of *λ* (Figs 4a and S3). We also examined the log-likelihood on unseen test data and confirmed that the estimation did not suffer from overfitting. As expected, too large a value of *λ* resulted in a failure to fit the data, because all parameters were estimated to be zero (Supplementary Fig. S4). Values of *λ* from 0.1 to 10 yielded high log-likelihood values, implying potential good inference; however, too small a value of *λ* (*λ* = 0.1, 1) resulted in inference of overly redundant and biologically inconsistent models (e.g., gene *Z* simultaneously autoactivated and autorepressed gene *Z*) (Supplementary Fig. S4). Thus, we rejected these models (Supplementary Fig. S4). Consequently, the results at *λ* = 3,10 were systematically selected as candidates for the inferred model.

**Figure 5:**
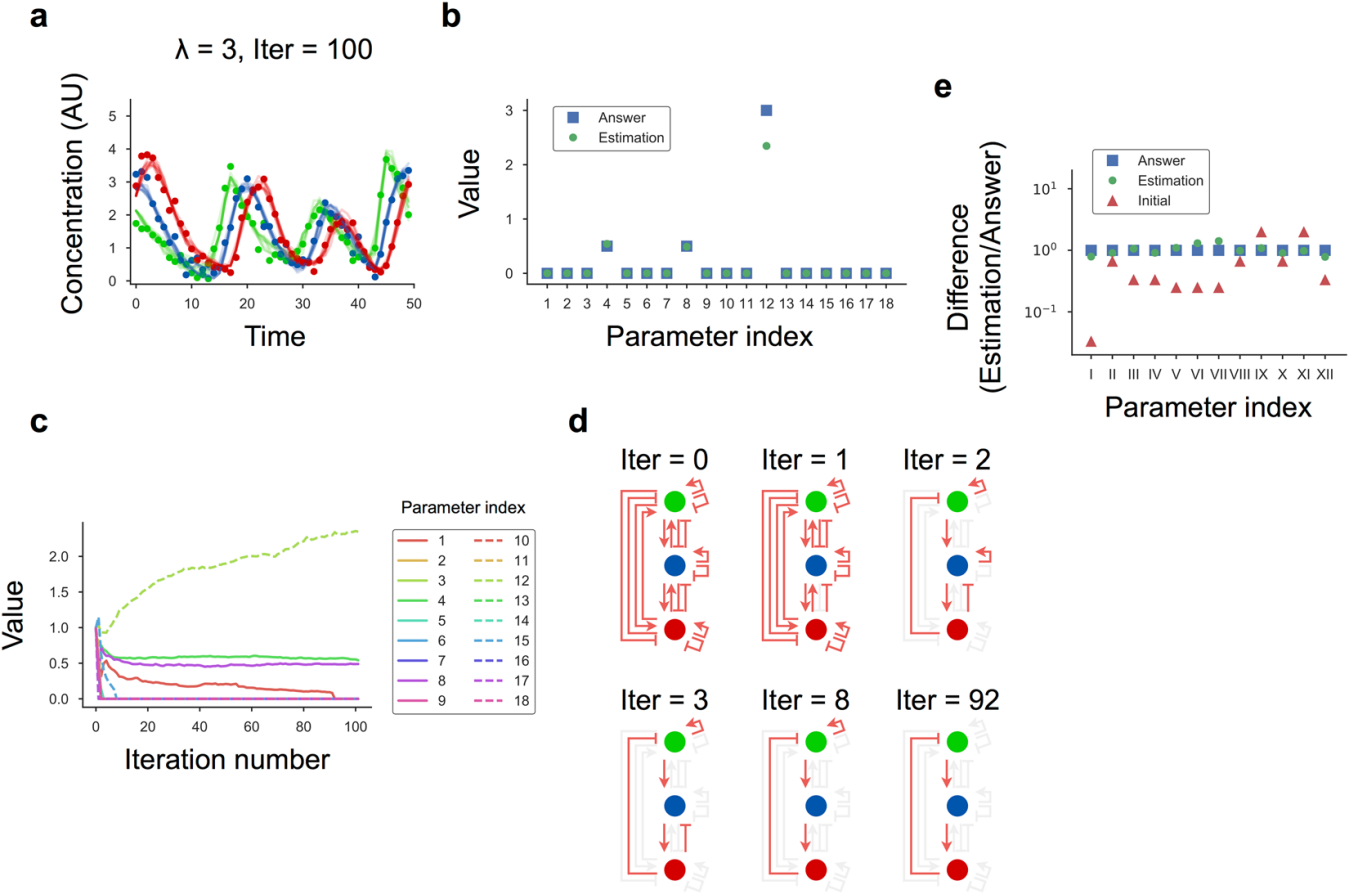
Data-driven inference of a three-component oscillator model. (**a**) Estimated states after 100 iterations of the algorithm with *λ* = 3. Each dot represents artificial data (Fig. 2b), and lines indicate the estimated trajectories. (**b**) Values of the association constant after 100 iterations of the algorithm with *λ* = 3. Each parameter index corresponds to the reaction number (Fig. 3). (**c**) Values of association constants in the model plotted as a function of the iteration number. (**d**) Schematic representation of the inferred network. The red arrows represent effective paths where the association constant has a nonzero value, whereas light-gray arrows are paths that have no regulatory activities, because the association constant is zero. (**e**) A difference in the parameter values between the estimated and correct values is shown as a ratio.

At *λ* = 3, the estimated states based on the inferred model were consistent with the data (Fig. 5a). In the inferred model, three association constants of 18 had nonzero values, indicating that only these three regulatory paths were crucial to reproduce the data (Fig. 5b). The paths consisted of activation of gene *Y* by gene *X*, activation of gene *Z* by gene *Y*, and repression of gene *X* by gene *Z*, which were equivalent to the true network (Fig. 2a). Removal of paths from the redundant model during iterations of the algorithm occurred at several steps rather than at a single step (Fig. 5c and d). We also confirmed that model parameters other than the association constants, such as degradation rate constants and Hill coefficients, were also successfully estimated (Fig. 5e). The same network model was inferred at *λ* = 10 (Supplementary Fig. S5). By contrast, the dynamics of the removal of paths from the redundant model were highly different from those at *λ* = 3. Overall, we demonstrated that the EM-PS-Lasso algorithm enabled both estimation of model parameters and inference of network topology. Furthermore, our results indicated that rich information regarding network topology was embedded in single-cell time-course data, even when the data were highly dynamic, nonlinear, and heterogeneous.

In general, the number of effective paths with nonzero association constants decreased as *λ* increased (Fig. 4b). Note that there was an apparent increase in the number of estimated paths at *λ* = 30. Indeed, most of the estimated paths had nonzero but extremely small values of association constants and had practically little effect on system behavior. This issue could be overcome by defining a threshold for the parameter value and/or for a degree of response to parameter changes (i.e., sensitivity analysis).

### Inferring the number of components in the network

**Figure 6:**
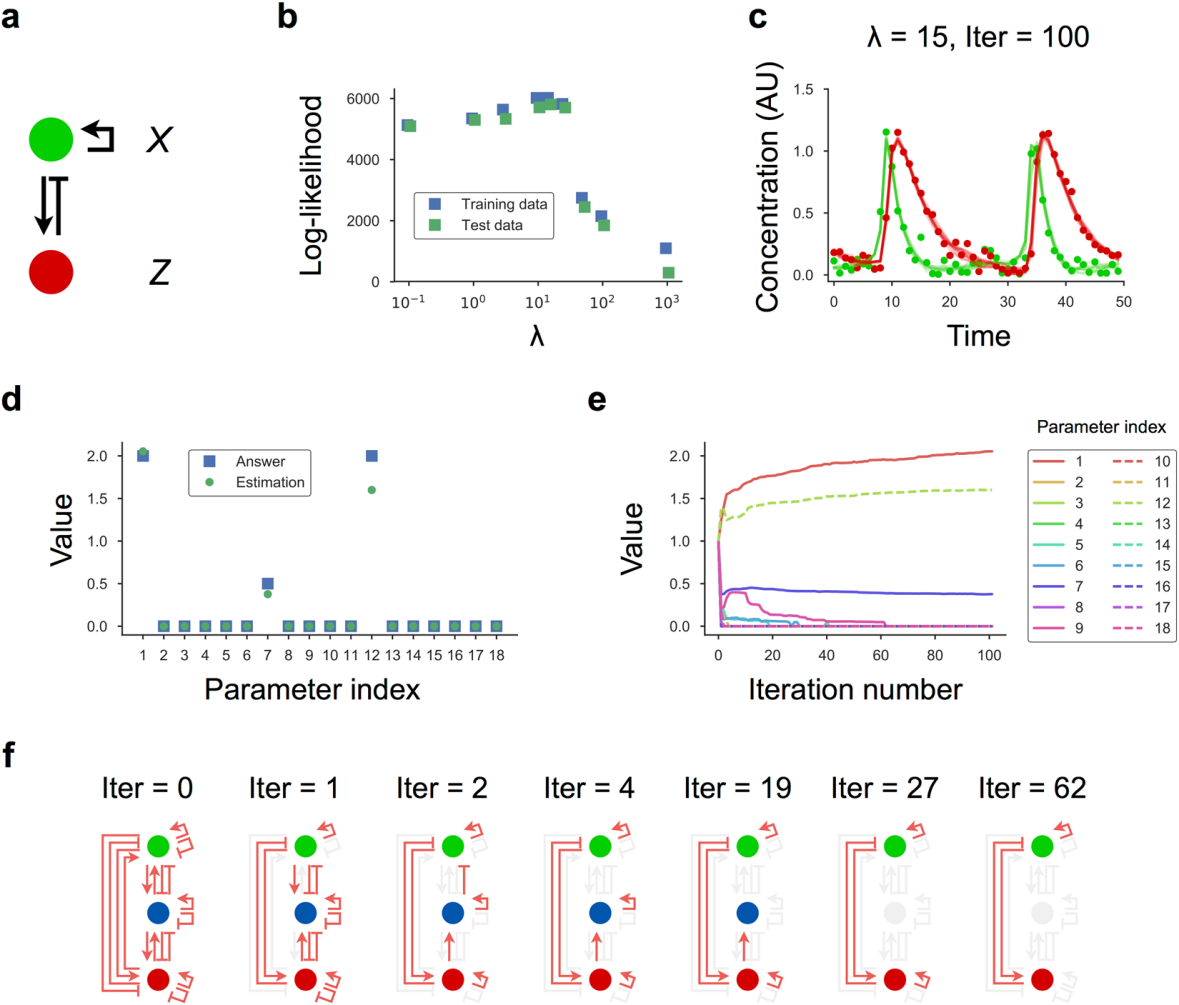
Inferring the number of components in the network. (**a**) Schematic of a two-component oscillator model. (**b**) Models were inferred using the EM-PS-Lasso algorithm with different values of *λ*. Log-likelihood values of the models at iteration number 100 are shown as a function of *λ*. (**c**) Consistency of the estimated states with the data. Each dot represents artificial data generated from the two-component oscillatory model. Lines denote trajectories sampled from the inferred model. (**d**) Values of the association constant after 100 iterations of the algorithm. Each parameter index corresponds to the reaction number (Fig. 3). (**e**) Values of association constants in the model plotted as a function of the iteration number of the EM-PS-Lasso algorithm. (**f**) Schematic representation of the inferred network. The red and light-gray arrows represent the effective paths and eliminated paths, respectively. Note that gene *Y* is not involved in system behavior, because all paths related to gene *Y* had no regulatory activity after iteration number 27.

In our analysis, we assumed that the number of genes constituting the network was known, whereas their regulatory relationships were unknown. However, it is more common that neither factor is known. Therefore, we examined whether the algorithm could infer both the number of components and network topology. Again, we generated an artificial dataset using a network consisting of two genes (Fig. 6a and Supplementary Information) showing oscillatory dynamics with appropriate parameters. We also prepared a redundant model (equivalent to that in Fig. 3) consisting of three components rather than two, because we assumed that we had no prior knowledge regarding the number of components in the network. Using the artificial data and redundant model, we performed model inference of the gene regulatory network via the EM-PS-Lasso algorithm and evaluated the log-likelihood of the inferred models, finding that the model with *λ* = 15 showed the highest log-likelihood value (Fig. 6b) and was consistent with the data (Fig. 6c). Next, we evaluated the values of the association constant for all regulatory paths in the redundant model. The paths in the redundant model were removed in several steps during iterations of the algorithm (Fig. 6d and e), with three association constants of 18 eventually found to have nonzero values (Fig. 6f). The paths remaining in the model described autoactivation of gene *X*, activation of gene *Z* by gene *X*, and repression of gene *X* by gene *Z*. All regulatory paths related to gene *Y* had no activity, indicating the absence of gene *Y* in the network model. Therefore, the inferred model practically consisted of two genes and three regulatory paths (Fig. 6e, right) and was completely equivalent to the true network (Fig. 6a). Overall, these results revealed that the EM-PS-Lasso algorithm was able to infer not only the regulatory paths but also the number of components in the model.

## Discussion

The concept of data-driven inference and analysis of biochemical networks has gained attention in computational systems biology and biophysics. However, this remains a difficult task due to the highly nonlinear nature of biological systems. Here, we proposed an EM algorithm-based method combining a particle smoother and sparse regularization to enable data-driven and systematic inference of nonlinear biochemical network models. Our method was successfully applied to construct mathematical models showing oscillations, which is one of the stereotypical nonlinear behaviors. Furthermore, because the elemental reaction in our modeling is described by a Hill function commonly used to express various types of biochemical reactions, our method can be directly applied to a wide range of networks, including transcriptional control, signal transduction, and metabolic regulation.

In this study, we focused on the fact that a regulatory path can be negligible when the association constant in the Hill function describing the path is equal to zero. Penalizing the association constants using Lasso resulted in elimination of unnecessary paths in the redundant model and enabled inference of the network topology. The proposed algorithm might also be useful when the model is described by other schemes, such as mass action kinetics, because the biological meaning of the association constant is straightforward. In such a system, the reaction is negligible when the association rate constant (*k*_on_) in the mass action kinetics is estimated at zero. Therefore, the algorithm would be applicable to the mass action-based model with only a slight modification, where the association rate constant instead of the association constant is subjected to regularization.

Although Lasso is a simple yet powerful technique that provides a sparse solution, there are also other methods for sparse regularization. For example, automatic relevance determination and Bayesian masking are superior to Lasso in terms of sparsity-shrinkage tradeoff^40,41^, although we did not use these techniques in the present study because of their slow convergence. Another promising approach to regularization is Group Lasso^24,42,43^, which can provide a sparse solution at the grouped variable level. Recently, a problem involving insulation of network activity attracted interest, and a condition that insulates the activity of a sub-network from the overall network was also studied^44^. The prominent feature of Group Lasso, where the sparse solution is given at the group level, might make it compatible with this problem.

Different system configurations often produce qualitatively similar behaviors^45^. For example, ~10 types of different synthetic circuits reportedly generate “oscillatory” dynamics^46^. Therefore, our finding that the network can be reconstructed based solely on time-course data might be surprising. Although it seems difficult to strictly define a condition that achieves the most effective inference, our results suggest that time-course data and possibly their associated noise^47^ contain rich information and would be sufficient to reconstruct the regulatory network. Methods for data-driven analysis will become increasingly important as the number of various experimental technologies, including super-multiplexed color live-cell imaging^48^, continue to rapidly progress. The present study provides a general framework for analyzing the intersections of nonlinear biochemical systems, model inference, and single-cell time-course data analysis in a data-driven manner.

## Materials and Methods

### Nonlinear state space model

We introduce a nonlinear state space model, which is given by

4 $$\begin{array}{rcl}{{\boldsymbol{x}}}_{t} & = & {\boldsymbol{f}}({{\boldsymbol{x}}}_{t-1})+{{\boldsymbol{v}}}_{t}\\ {{\boldsymbol{y}}}_{t} & = & {\boldsymbol{h}}({{\boldsymbol{x}}}_{t})+{{\boldsymbol{w}}}_{t}\end{array}$$

where ***x*** is a *k*-dimensional vector consisting of state variables, and ***y*** denotes an *l*-dimensional vector representing measurements of ***x***. Functions ***f*** and ***h*** are nonlinear functions describing the evolution of the system and measurement process, respectively. Vectors ***v***_*t*_ = {***v***_*t*,*i*_} (*i* = 1, …, *k*) and ***w***_*t*_ = {***w***_*t*,*j*_} (*j* = 1, …, *l*) denote system noise and measurement noise, respectively, where we assumed that they followed a Gaussian distribution: *v*_*t*,*i*_ ~ *N* (0, (*σ*_*i*_)^2^), *w*_*t*,*j*_ ~ *N* (0, (*η*_*j*_)^2^). Initial values of the state are given by *x*_0,*i*_ ~ *N* (*μ*_*i*_, (*γ*_*i*_)^2^). We used standard ODEs to model the biochemical reaction network of interest as *d****x***/*dt* = ***g*** (***x***, ***θ***_*sys*_), where, in general, ***g*** is a nonlinear function consisting of arbitrary equations, such as the Hill equation, and ***θ***_*sys*_ indicates model parameters. Function ***f*** can be calculated by numerically integrating the equations as ${\boldsymbol{f}}({{\boldsymbol{x}}}_{t-1})={{\boldsymbol{x}}}_{t-1}+\,{\int }_{t-1}^{t}\,{\boldsymbol{g}}({{\boldsymbol{x}}}_{\tau },{{\boldsymbol{\theta }}}_{sys})d\tau$. Numerical integration of ODEs was performed using routines implemented in the scipy.integrate package (https://docs.scipy.org/doc/scipy/reference/integrate.html) as described previously^49^. In the present study, we used a linear function for ***h*** (***h***(***x***) = *α****x***) for simplicity, where *α* = 1 unless otherwise explicitly indicated. Given dataset $Y=\{{Y}^{(a)}\}=\{{Y}^{(1)},{Y}^{(2)},\ldots ,{Y}^{(A)}\}=\{{{\boldsymbol{y}}}_{1:T}^{(1)},{{\boldsymbol{y}}}_{1:T}^{(2)},\ldots {{\boldsymbol{y}}}_{1:T}^{(A)}\}$, where *a* is an index of each cell and *Y*^(*a*)^ represents the single-cell time-course data, estimation of ${\boldsymbol{\theta }}=\{{{\boldsymbol{\theta }}}_{sys},{\boldsymbol{\sigma }},{\boldsymbol{\eta }},{\boldsymbol{\mu }},{\boldsymbol{\gamma }}\},({\boldsymbol{\sigma }}=\{{\sigma }_{i}\},{\boldsymbol{\eta }}=\{{\eta }_{j}\},{\boldsymbol{\mu }}=\{{\mu }_{i}^{(a)}\},{\boldsymbol{\gamma }}\,=$ $\{{\gamma }_{i}\}\,(i=1,\ldots ,k,j=1,\ldots ,l,a=1,\ldots ,A))$ can be accomplished by maximizing the log-likelihood of the model. Note that only ***μ*** is dependent on the cell index *a* to describe cell-to-cell variability of initial states.

### EM-PS algorithm for parameter estimation

Maximum likelihood estimation of ***θ*** can be accomplished by maximizing log-likelihood $\mathrm{ln}\,p(Y|{\boldsymbol{\theta }})=\,\mathrm{ln}\,\sum _{X}\,p(Y|X,{\boldsymbol{\theta }})p(X|{\boldsymbol{\theta }})$, which requires intractable integration with respect to state variables $X=\{{X}^{(a)}\}=\{{X}^{(1)},{X}^{(2)},\ldots ,{X}^{(A)}\}=\{{{\boldsymbol{x}}}_{1:T}^{(1)},{{\boldsymbol{x}}}_{1:T}^{(2)},\ldots ,{{\boldsymbol{x}}}_{1:T}^{(A)}\}$. Therefore, we used an EM algorithm to find maximum likelihood estimates of ***θ***. The EM algorithm was run by iterating steps E (expectation) and M (maximization), which are defined as

5 $$\begin{array}{rcl}Q({\boldsymbol{\theta }},{{\boldsymbol{\theta }}}^{({\rm{old}})}) & = & {\langle \mathrm{ln}p(X,Y|{\boldsymbol{\theta }})\rangle }_{p(X|Y,{{\boldsymbol{\theta }}}^{({\rm{old}})})}\\ {{\boldsymbol{\theta }}}^{({\rm{new}})} & = & {\rm{\arg }}\,\mathop{{\rm{\max }}}\limits_{{\boldsymbol{\theta }}}Q\,({\boldsymbol{\theta }},{{\boldsymbol{\theta }}}^{({\rm{old}})})\end{array}$$

respectively. Given that the E step is analytically intractable, because it requires the probability distribution of the time series at all time points, we numerically approximated *p*(*X*|*Y*, ***θ***) using a particle smoother as previously reported^31,32^. Briefly, the particle smoother algorithm approximates the distribution as an ensemble of particles:

6 $$p({X}^{(a)}|{Y}^{(a)},{\boldsymbol{\theta }})=\sum _{p=1}^{P}\,{\beta }^{(a,p)}\delta ({X}^{(a)}-{X}^{(a,p)}),\sum _{p=1}^{P}{\beta }^{(a,p)}=1,{\beta }^{(a,p)}\ge 0$$

where *P* is the number of particles, *X*^(*a*,*p*)^ indicates a trajectory of the *p*^th^ particle sampled by the algorithm for data *Y*^(*a*)^, *β*^*(a*,*p*)^ represents the weight of the particle, and 𝛿 is Dirac’s delta. This weight is given as ${\beta }^{(a,p)}={l}^{(a,p)}/\sum _{p}{l}^{(a,p)}$, where *l*^(*a*,*p*)^ = *p* (*Y*^(*a*,*p*)^|*X*^(*a*,*p*)^) denotes the likelihood of the particle. The calculation was performed using the pyParticleEst package^50^. Finally, the log-likelihood estimate was obtained by averaging over the particles: ${\rm{l}}{\rm{n}}\,{L}^{(a)}({\boldsymbol{\theta }})={\rm{l}}{\rm{n}}\,(\frac{1}{P}\sum _{p}\,{l}^{(a,p)})$. Note that $\mathrm{ln}\,p(X|Y,{\boldsymbol{\theta }})$ can be written as $\mathrm{ln}\,p(X|Y,{\boldsymbol{\theta }})=\sum _{a}\,\mathrm{ln}\,p({X}^{(a)}|{Y}^{(a)},{\boldsymbol{\theta }})$, because different time-course data are independent. Thus, the E step can be completed using the following approximation:

7 $$\begin{array}{ccc}Q({\boldsymbol{\theta }},{{\boldsymbol{\theta }}}^{({\rm{o}}{\rm{l}}{\rm{d}})}) & = & \sum _{a=1}^{A}\,{\langle {\rm{l}}{\rm{n}}p({X}^{(a)},{Y}^{(a)}|{\boldsymbol{\theta }})\rangle }_{p({X}^{(a)}|{Y}^{(a)},{{\boldsymbol{\theta }}}^{({\rm{o}}{\rm{l}}{\rm{d}})})}\\  & = & \sum _{a=1}^{A}\sum _{p=1}^{P}{\beta }^{(a,p)}\,{\rm{l}}{\rm{n}}\,p({X}^{(a,p)},{Y}^{(a)}|{\boldsymbol{\theta }})\,\\  & = & \,\sum _{a=1}^{A}\sum _{p=1}^{P}\sum _{i=1}^{k}{\beta }^{(a,p)}(-\frac{1}{2}\,{\rm{l}}{\rm{n}}\,2\pi {({\gamma }_{i})}^{2}-\frac{{({x}_{0,i}^{(a,p)}-{\mu }_{i}^{(a)})}^{2}\,}{2{({\gamma }_{i})}^{2}})\\  &  & +\,\sum _{a=1}^{A}\sum _{p=1}^{P}\sum _{t\in T}\sum _{i=1}^{k}{\beta }^{(a,p)}(-\frac{1}{2}\,{\rm{l}}{\rm{n}}\,2\pi {({\sigma }_{i})}^{2}-\frac{{({x}_{t,i}^{(a,p)}-{f}_{i}({{\boldsymbol{x}}}_{t-1}^{(a,p)},{{\boldsymbol{\theta }}}_{sys}))}^{2}}{2{({\sigma }_{i})}^{2}})\\  &  & +\,\,\sum _{a=1}^{A}\sum _{p=1}^{P}\sum _{t\in T}\sum _{j=1}^{l}{\beta }^{(a,p)}(-\frac{1}{2}\,{\rm{l}}{\rm{n}}\,2\pi {({\eta }_{j})}^{2}-\frac{{({y}_{t,j}^{(a)}-{h}_{j}({{\boldsymbol{x}}}_{t}^{(a,p)}))}^{2}}{2{({\eta }_{j})}^{2}}).\end{array}$$

For the M step, we numerically maximized the *Q* function using the quasi-Newton method with respect to ***θ***_*sys*_, because, in general, *dQ*/*d****θ***_*sys*_ = 0 cannot be solved analytically. This optimization was performed using the L-BFGS-B function implemented in the scipy.optimize package (http://docs.scipy.org/doc/scipy/reference/optimize.html), with a non-negative constraint for the parameter values. Additionally, the equations for the derivative of *Q* with respect to ***σ***, ***η***, ***μ***, and ***γ*** are linear equations; therefore, updated values for the parameters were easily found. Note that we defined minimum values for ***γ***, because if the value is too small, sample impoverishment can occur^51^. We also defined maximum values for ***σ***, ***η*** in order to avoid overestimation of the noise that could cause meaningless inference.

### Artificial data generation

Artificial data were generated by numerically solving the model as nonlinear stochastic Langevin equations: *d****x***/*dt* = ***g***(***x***) + ***ξ***(*t*), where ***ξ***(*t*) is Gaussian noise with 〈*ξ*_*i*_(*t*) = 0〉 and 〈*ξ*_*i*_(*t*)*ξ*_*j*_ (*t*′)〉 = 2*Dδ*_*i*,*j*_*δ* (*t* − *t*′) with Kronecker’s *δ*_*i*,*j*_ and Dirac’s *δ*(*t*) distribution, where parameter *D* characterizes the amplitude of the noise. Computation was conducted using a stochastic Runge-Kutta algorithm^52^. The measurement process was simulated by adding Gaussian noise to each variable: *y*_*i*_ = *x*_*i*_ + *ηϕ*, where *ϕ* is a random number sampled from a standard normal distribution, and *η* characterizes the amplitude of the noise. Stochastic simulation was performed over the simulation period *T* = 400, and data points from *T* = 351 to *T* = 400 were collected at a time resolution of 1. The simulation was repeated 10 times to generate 10 independent time-course data that served as training data. Similarly, an additional 10 independent time-course data were generated and used as test data for validation. Details of the model equations and parameter values are described in the Supporting Information.

## Electronic supplementary material

Supplementary information

## References

[1] Ferrell JE, Machleder EM (1998). The biochemical basis of an all-or-none cell fate switch in Xenopus oocytes. Science.

[2] Xiong W, Ferrell JE (2003). A positive-feedback-based bistable ‘memory module’ that governs a cell fate decision. Nature.

[3] Arata Y (2016). Cortical Polarity of the RING Protein PAR-2 is Maintained by Exchange Rate Kinetics at the Cortical-Cytoplasm Boundary. Cell Rep..

[4] Tay S (2010). Single-cell NF-κB dynamics reveal digital activation and analogue information processing. Nature.

[5] Lev Bar-Or R (2000). Generation of oscillations by the p53-Mdm2 feedback loop: a theoretical and experimental study. Proc. Natl. Acad. Sci. USA.

[6] Shinohara H (2014). Positive feedback within a kinase signaling complex functions as a switch mechanism for NF-κB activation. Science.

[7] Alon U (2007). Network motifs: theory and experimental approaches. Nat. Rev. Genet..

[8] Uda S (2013). Robustness and compensation of information transmission of signaling pathways. Science.

[9] Roob E, Trendel N, Rein ten Wolde P, Mugler A (2016). Cooperative Clustering Digitizes Biochemical Signaling and Enhances its Fidelity. Biophys. J..

[10] Stoeger T, Battich N, Pelkmans L (2016). Passive Noise Filtering by Cellular Compartmentalization. Cell.

[11] Kellogg RA, Tay S (2015). Noise Facilitates Transcriptional Control under Dynamic Inputs. Cell.

[12] Waltermann C, Klipp E (2011). Information theory based approaches to cellular signaling. Biochim. Biophys. Acta.

[13] Kholodenko BN, Demin OV, Moehren G, Hoek JB (1999). Quantification of short term signaling by the epidermal growth factor receptor. J. Biol. Chem..

[14] Schoeberl B, Eichler-Jonsson C, Gilles ED, Müller G (2002). Computational modeling of the dynamics of the MAP kinase cascade activated by surface and internalized EGF receptors. Nat. Biotechnol..

[15] Sasagawa S, Ozaki Y, Fujita K, Kuroda S (2005). Prediction and validation of the distinct dynamics of transient and sustained ERK activation. Nat. Cell Biol..

[16] Iwamoto K, Shindo Y, Takahashi K (2016). Modeling Cellular Noise Underlying Heterogeneous Cell Responses in the Epidermal Growth Factor Signaling Pathway. PLoS Comput. Biol..

[17] Hempel S, Koseska A, Nikoloski Z, Kurths J (2011). Unraveling gene regulatory networks from time-resolved gene expression data – a measures comparison study. BMC Bioinformatics.

[18] Omony J (2014). Biological Network Inference: A Review of Methods and Assessment of Tools and Techniques. Annu. Res. Rev. Biol..

[19] Omranian N, Eloundou-Mbebi JMO, Mueller-Roeber B, Nikoloski Z (2016). Gene regulatory network inference using fused LASSO on multiple data sets. Sci. Rep..

[20] Yan B (2017). An integrative method to decode regulatory logics in gene transcription. Nat. Commun..

[21] Forrest ARR (2014). A promoter-level mammalian expression atlas. Nature.

[22] Lundby A (2012). Quantitative maps of protein phosphorylation sites across 14 different rat organs and tissues. Nat. Commun..

[23] Toni T, Welch D, Strelkowa N, Ipsen A, Stumpf MPH (2009). Approximate Bayesian computation scheme for parameter inference and model selection in dynamical systems. J. R. Soc. Interface.

[24] Wu H, Lu T, Xue H, Liang H (2014). Sparse additive ordinary differential equations for dynamic gene regulatory network modeling. J. Am. Stat. Assoc..

[25] Chen, S., Shojaie, A. & Witten, D. M. Network Reconstruction From High Dimensional Ordinary Differential Equations. *J*. *Am*. *Stat*. *Assoc*., 10.1080/01621459.2016.1229197 (2016). 10.1080/01621459.2016.1229197 PMC5880569 29618851

[26] Brunton SL, Proctor JL, Kutz JN (2016). Discovering governing equations from data: Sparse identification of nonlinear dynamical systems. Proc. Natl. Acad. Sci. USA.

[27] Oates CJ (2014). Causal network inference using biochemical kinetics. Bioinformatics.

[28] Mangan NM, Brunton SL, Proctor JL, Kutz JN (2016). Inferring Biological Networks by Sparse Identification of Nonlinear Dynamics. IEEE Trans. Mol. Biol. Multi-Scale Commun..

[29] Spiller DG, Wood CD, Rand DA, White MRH (2010). Measurement of single-cell dynamics. Nature.

[30] Url S, Statistics G (1996). Monte Carlo Filter and Smoother for Non-Gaussian Nonlinear State Space Models Genshiro Kitagawa. J. Comput. Graph. Stat..

[31] Andrieu C, Doucet A, Singh SS, Tadic VB (2004). Particle methods for change detection, system identification, and control. Proc. IEEE.

[32] Kondo Y, Kaneko K, Ishihara S (2013). Identifying dynamical systems with bifurcations from noisy partial observation. Phys. Rev. E.

[33] Thieffry D, Huerta AM, Pérez-Rueda E, Collado-Vides J (1998). From specific gene regulation to genomic networks: a global analysis of transcriptional regulation in *Escherichia coli*. BioEssays.

[34] Tegner J, Yeung MKS, Hasty J, Collins JJ (2003). Reverse engineering gene networks: Integrating genetic perturbations with dynamical modeling. Proc. Natl. Acad. Sci. USA.

[35] Cai X, Bazerque JA, Giannakis GB (2013). Inference of Gene Regulatory Networks with Sparse Structural Equation Models Exploiting Genetic Perturbations. PLoS Comput. Biol..

[36] Jia B, Wang X (2014). Regularized EM algorithm for sparse parameter estimation in nonlinear dynamic systems with application to gene regulatory network inference. EURASIP J. Bioinformatics Syst. Biol..

[37] Hasegawa T, Yamaguchi R, Nagasaki M, Miyano S, Imoto S (2014). Inference of gene regulatory networks incorporating multi-source biological knowledge via a state space model with L1 regularization. Plos One.

[38] Tibshirani R (1996). Regression Shrinkage and Selection via the Lasso. J. R. Stat. Soc. Ser. B.

[39] Alon, U. *An Introduction to Systems Biology: Design Principles of Biological Circuits*. (Chapman and Hall/CRC 2006).

[40] Aravkin A, Burke JV, Chiuso A, Pillonetto G (2014). Convex vs Non-Convex Estimators for Regression and Sparse Estimation: the Mean Squared Error Properties of ARD and GLasso. J. Mach. Learn. Res..

[41] Kondo Y, Hayashi K, Maeda S (2016). Bayesian Masking: Sparse Bayesian Estimation with Weaker Shrinkage Bias. Proc. Mach. Learn. Res..

[42] Yuan M, Lin Y (2006). Model selection and estimation in regression with grouped variables. J. R. Stat. Soc. Ser. B.

[43] Yang G, Wang L, Wang X (2017). Reconstruction of Complex Directional Networks with Group Lasso Nonlinear Conditional Granger Causality. Sci. Rep..

[44] Atay O, Doncic A, Skotheim JM (2016). Switch-like Transitions Insulate Network Motifs to Modularize Biological Networks. Cell Syst..

[45] Bansal M, Belcastro V, Ambesi-Impiombato A, di Bernardo D (2007). How to infer gene networks from expression profiles. Mol. Syst. Biol..

[46] Purcell O, Savery NJ, Grierson CS, Bernardo M (2010). A comparative analysis of synthetic genetic oscillators. J. R. Soc. Interface.

[47] Lipinski-Kruszka J, Stewart-Ornstein J, Chevalier MW, El-Samad H (2015). Using Dynamic Noise Propagation to Infer Causal Regulatory Relationships in Biochemical Networks. ACS Synth. Biol..

[48] Wei L (2017). Super-multiplex vibrational imaging. Nature.

[49] Shindo Y (2016). Conversion of graded phosphorylation into switch-like nuclear translocation via autoregulatory mechanisms in ERK signalling. Nat. Commun..

[50] Nordh, J. pyParticleEst: A Python Framework for Particle-Based Estimation Methods. *J*. *Stat*. *Softw*. **78** (2017).

[51] Arulampalam MS, Maskell S, Gordon N, Clapp T (2002). A tutorial on particle filters for online nonlinear/non-Gaussian Bayesian tracking. IEEE Trans. Signal Process..

[52] Honeycutt RL (1992). Stochastic Runge-Kutta algorithms. I. White noise. Phys. Rev. A.

