# Supplementary material for: Inferring a nonlinear biochemical network model from a heterogeneous single-cell time course data
Source: Sci Rep. 2018 May 1;8:6790. doi: 10.1038/s41598-018-25064-w (PMC5931614; doi:10.1038/s41598-018-25064-w)
Supplement: Supplementary file 1 — Supplementary information [file 41598_2018_25064_MOESM1_ESM.pdf]

Supplementary Information

Inferring a nonlinear biochemical network model from a heterogeneous single-cell time course data

Yuki Shindo[1], Yohei Kondo[2], and Yasushi Sako[1]

[1] Cellular Informatics Laboratory, RIKEN, Wako, Saitama 351-0198, Japan

[2] Graduate School of Informatics, Kyoto University, Sakyo-ku, Kyoto 606-8501, Japan

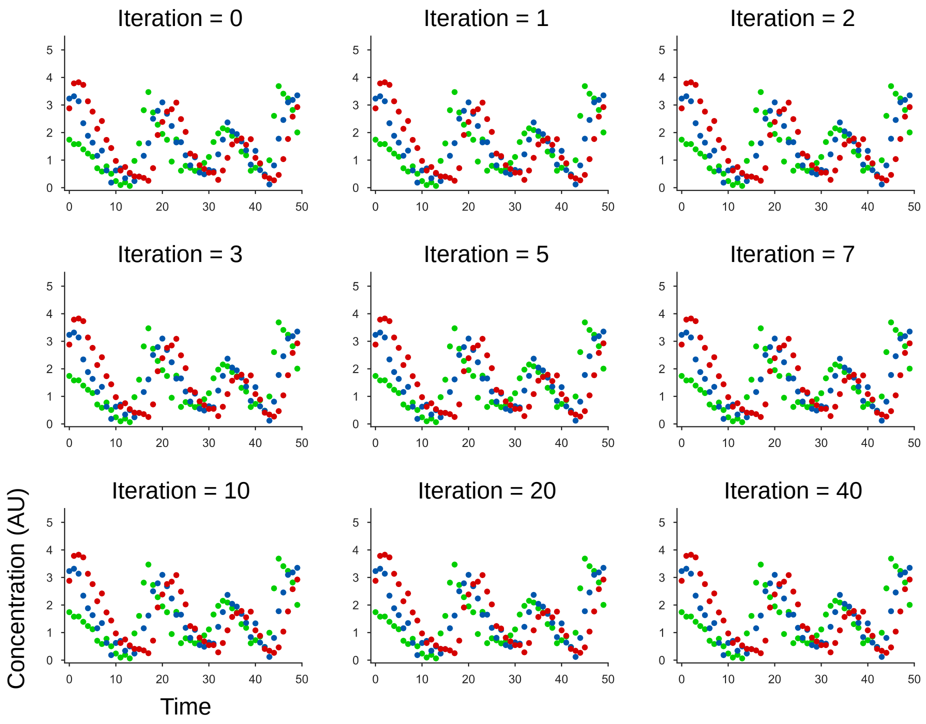

Supplementary Figure S1 Improvement of estimated states during iterations of the algorithm. Each dot represents the (artificial) measurement data and lines denote the trajectories sampled by the particle smoother. The result of the estimation for simulation #1 with Initial #1 (Supplementary Table S1) as initial values is shown.

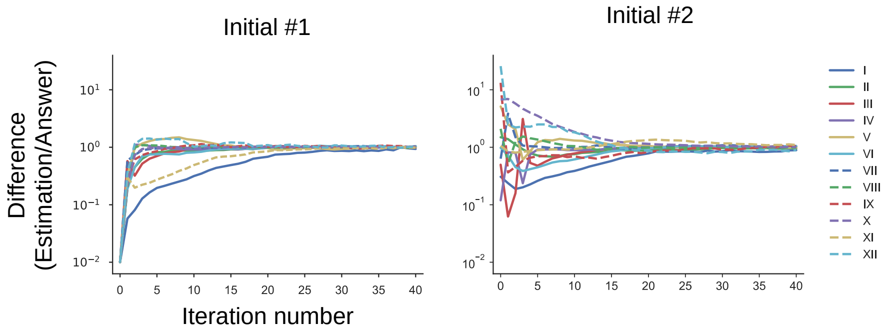

Index	Parameter
I	s
II	k_{-1}
III	a_2
IV	K_2
V	n_2
VI	k_{-2}
VII	a_3
VIII	K_3
IX	n_3
X	k_{-3}
XI	K_1
XII	n_1

Supplementary Figure S2 Convergence of each model parameter during iterations of the EM-PS algorithm.

The ratio of estimated parameter values to correct values is plotted as a function of the iteration number. The results of the estimation for two sets of initial values (Supplementary Table S1) are shown.

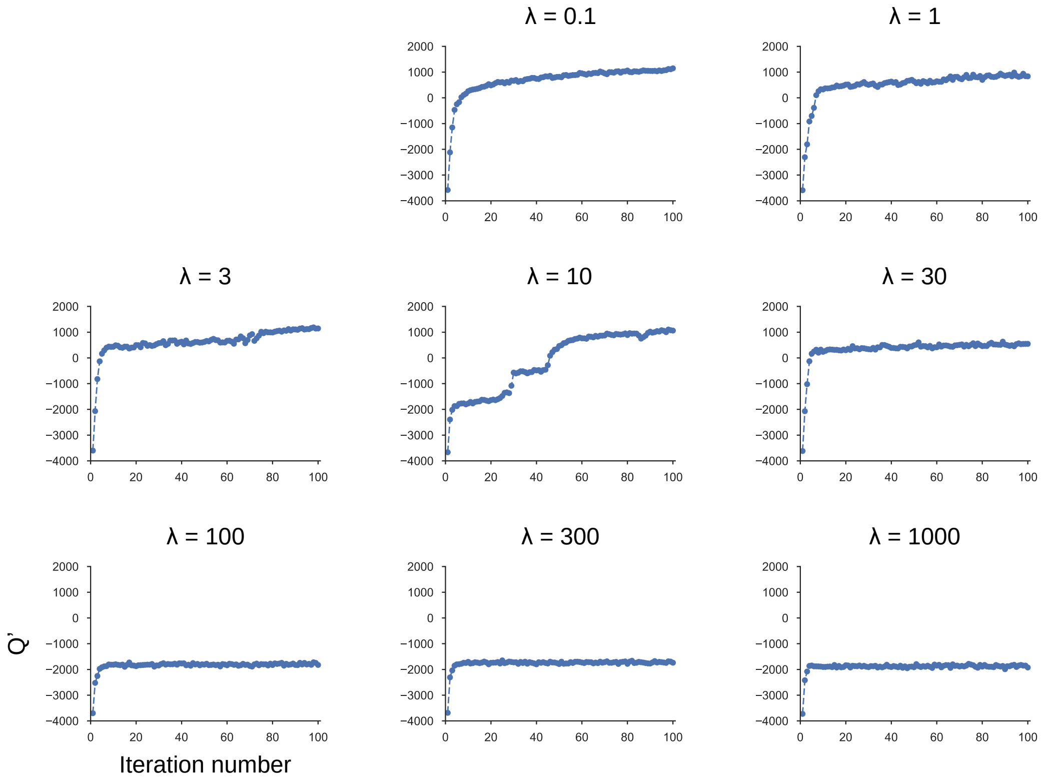

Supplementary Figure S3 Confirmation of convergence of the EM-PS-Lasso algorithm. Values of Q' at different values of λ are shown as a function of the iteration number.

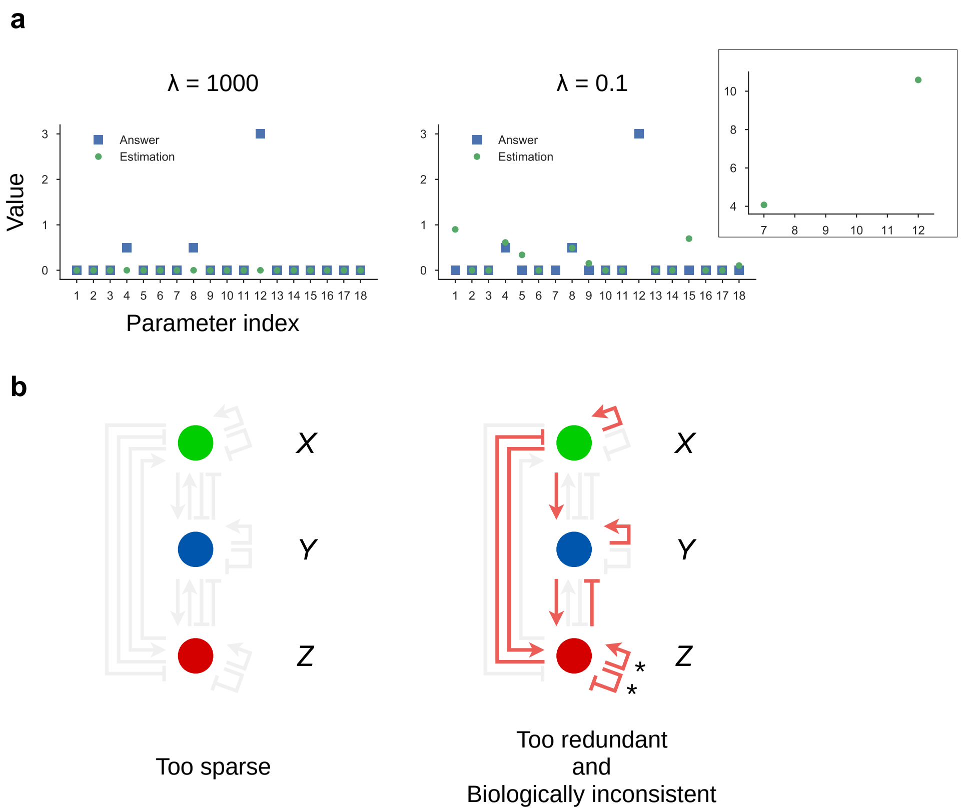

Supplementary Figure S4 Model inference using the EM-PS-Lasso algorithm with different values of λ.

(**a**) Estimated values of association constants after 100 iterations of the algorithm with $\lambda = 1000$ or $\lambda = 0.1$. The inset is the same plot focused on estimated values for parameter indices of 7 and 12 that showed large values compared to other parameters and did not fit in the main plot. (**b**) Schematic representation of the inferred model. The red arrows represent effective paths where the association constant has a nonzero value, whereas light-gray arrows are paths that have no regulatory activities, because the association constant is zero. All parameters decreased to zero with $\lambda = 1000$, while many parameters remained nonzero with $\lambda = 0.1$, resulting in too sparse or too redundant a model, respectively. In addition, the network structure is biologically inconsistent (asterisks) when $\lambda = 0.1$, leading to rejection of the model.

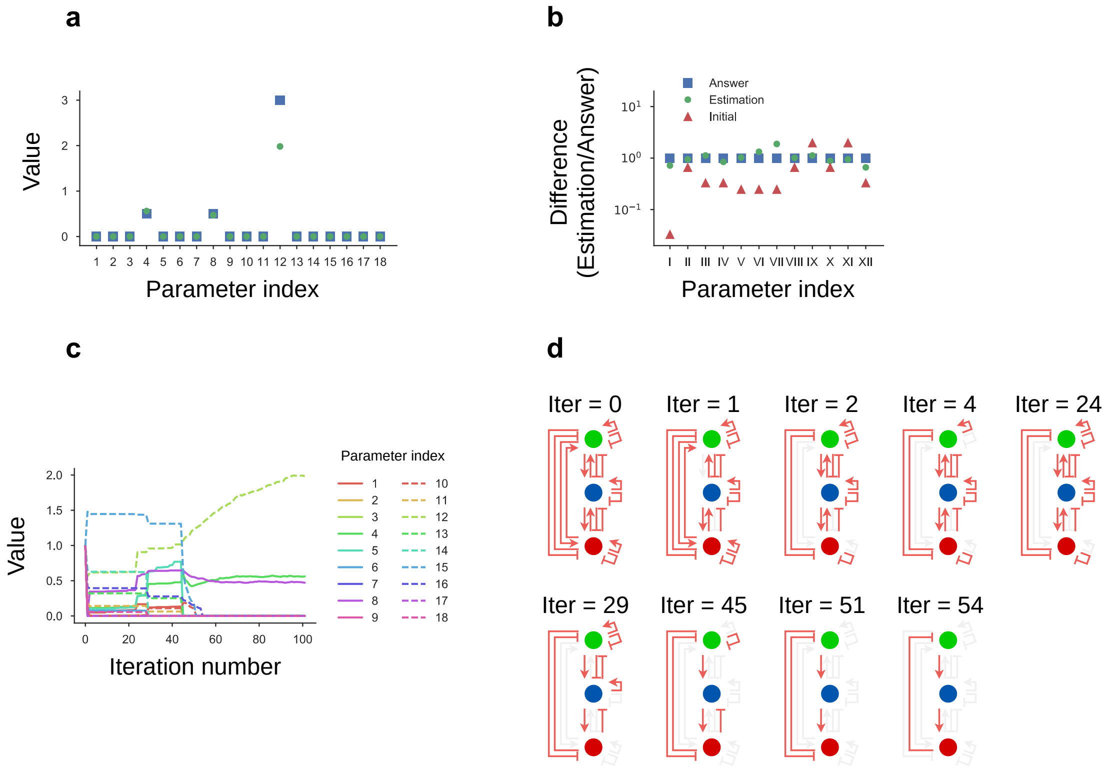

Supplementary Figure S5 Learning three-component oscillator model using the EM-PS-Lasso algorithm with $\lambda = 10$.

(**a**) Values of the association constant after 100 iterations of the algorithm are shown. Each parameter index corresponds to the reaction number (Fig. 3). (**b**) A difference in the parameter values between the estimated and correct values is shown as a ratio. (**c**) Values of association constants in the redundant model plotted as a function of the iteration number. (**d**) Schematic representation of the inferred model at each iteration number. The red and light-gray arrows represent the effective paths and eliminated paths, respectively.

Supplementary Materials and Methods

Three-component oscillator model

Schematic of the model is shown in Fig. 2A. Model equations were as follows:

$$\frac{d[X]}{dt} = s\frac{1}{1 + (K_1[Z])^{n_1}} - k_{-1}[X]$$

$$\frac{d[Y]}{dt} = a_2\frac{(K_2[X])^{n_2}}{1 + (K_2[X])^{n_2}} - k_{-2}[Y]$$

$$\frac{d[Z]}{dt} = a_3\frac{(K_3[Y])^{n_3}}{1 + (K_3[Y])^{n_3}} - k_{-3}[Z]$$

where parameter values were $s = 3.0$, $K_1 = 3.0, n_1 = 4.0$, $k_{-1} = 0.15$, $a_2 = 1.5$, $K_2 = 0.5$, $n_2 = 4.0$, $k_{-2} = 0.3$, $a_3 = 1.5$, $K_3 = 0.5$, $n_3 = 4.0$, $k_{-3} = 0.3$. Parameters and initial values used to generate the artificial data were $D = 5.0 \times 10^{-3}$, $\eta = 0.2$ and $[X_0] = [Y_0] = [Z_0] = 0.0$, respectively.

Two-component oscillator model

Schematic of the model is shown in Fig. 6A. Model equations were as follows:

$$\frac{d[X]}{dt} = \left(s + a_{11}\frac{(K_{11}[X])^{n_1}}{1 + (K_{11}[X])^{n_1}}\right)\frac{1}{1 + (K_{-21}[Z])^{n_2}} - k_{-1}[X]$$

$$\frac{d[Z]}{dt} = a_{12}\frac{(K_{12}[X])^{n_1}}{1 + (K_{12}[X])^{n_1}} - k_{-2}[Z]$$

where parameter values were $s = 0.03$, $a_{11} = 3.0$, $K_{11} = 2.0$, $n_1 = 2.0$, $k_{-1} = 1.0$, $a_{12} = 3.0$, $K_{12} = 0.5$, $n_2 = 2.0$, $k_{-2} = 0.2$, $K_{-21} = 2.0$. Parameters and initial values used for artificial data generation were $D = 5.0 \times 10^{-5}, \eta = 0.06$ and $[X_0] = [Z_0] = 0.0$, respectively.

Redundant model

Schematic of the model is shown in Fig. 3. Model equations were as follows:

$$\frac{d[X_1]}{dt} = \left(s + \sum_{j=1}^{3} a_{j1} \frac{\left(K_{j1}[X_j]\right)^{n_j}}{1 + \left(K_{j1}[X_j]\right)^{n_j}} \right) \cdot \prod_{j=1}^{3} \frac{1}{1 + \left(K_{-j1}[X_j]\right)^{n_j}} - k_{-1}[X_1]$$

$$\frac{d[X_2]}{dt} = \left(\sum_{j=1}^{3} a_{j2} \frac{\left(K_{j2}[X_j]\right)^{n_j}}{1 + \left(K_{j2}[X_j]\right)^{n_j}} \right) \cdot \prod_{j=1}^{3} \frac{1}{1 + \left(K_{-j2}[X_j]\right)^{n_j}} - k_{-2}[X_2]$$

$$\frac{d[X_3]}{dt} = \left(\sum_{j=1}^{3} a_{j3} \frac{\left(K_{j3}[X_j]\right)^{n_j}}{1 + \left(K_{j3}[X_j]\right)^{n_j}} \right) \cdot \prod_{j=1}^{3} \frac{1}{1 + \left(K_{-j3}[X_j]\right)^{n_j}} - k_{-3}[X_3]$$

where X_1, X_2, X_3 represent genes *X*, *Y*, and *Z*, respectively. Initial parameter values used for estimation are provided in Supplementary Tables 1–3 below.

Supplementary Table S1 Initial parameter values used for parameter estimation in Fig. 2.

Parameter	Initial #1	Initial #2
s	0.03	0.92
K_1	0.03	3.18
n_1	0.04	103
k_{-1}	0.0015	0.224
a_2	0.015	0.747
K_2	0.005	0.06
n_2	0.04	20.5
k_{-2}	0.003	0.294
a_3	0.015	0.952
K_3	0.005	1.05
n_3	0.04	53
k_{-3}	0.003	2.04
$\mu_i^{(a)}$	2.0	2.0
γ_i	0.2	0.2
σ_i	0.1	0.1
η_j	1.0	0.3
Minimum value of γ_i	0.01	0.01
Maximum value of σ_i	0.15	0.15
Maximum value of η_j	0.5	0.5

Note that $i = 1,2,3$; $j = 1,2,3$; $a = 1 \dots 10$. All units are arbitrary units of time or concentration. Initial #1 is 1/100 of the correct values and Initial #2 is randomly generated in the range of 1/30 to 30× the correct values.

Supplementary Table S2 Initial parameter values used for model inference in Fig. 5.

Parameter	Initial #1
s	0.1
k_{-i}	0.1
n_i	1.0
a_{kl}	0.1
K_{kl}	1.0
K_{-kl}	1.0
$\mu_i^{(a)}$	2.0
γ_i	0.2
σ_i	0.1
η_j	0.5
Minimum value of γ_i	0.01
Maximum value of σ_i	0.1
Maximum value of η_j	0.5

Note that $i = 1, 2, 3$; $j = 1, 2, 3$; $k = 1, 2, 3$; $l = 1, 2, 3$; $a = 1 \ldots 10$. All units are arbitrary units of time or concentration.

Supplementary Table S3 Initial parameter values used for model inference in Fig. 6.

Parameter	Initial #1
s	0.01
k_{-i}	0.1
n_i	1.0
a_{kl}	0.1
K_{kl}	1.0
K_{-kl}	1.0
$\mu_i^{(a)}$	0.5
γ_i	0.2
σ_i	0.025
η_j	0.1
Minimum value of γ_i	0.01
Maximum value of σ_i	0.025
Maximum value of η_j	0.1

Note that $i = 1, 2, 3$; $j = 1, 2$; $k = 1, 2, 3$; $l = 1, 2, 3$; $a = 1 \dots 10$. All units are arbitrary units of time or concentration.
